# OP02 Cost-Utility Of Geriatric Assessment And Management In Older Adults With Cancer: Model-Based Economic Evaluation

**DOI:** 10.1017/S0266462325100615

**Published:** 2025-12-29

**Authors:** Selai Akseer, Shant Torkom Yeretzian, Lusine Abrahamyan, Martine Puts, Mostafa Mohamed, Wee Kheng Soo, Shabbir Alibhai, Yeva Sahakyan

## Abstract

**Introduction:**

Geriatric assessment and management (GAM) is a guideline-recommended strategy for optimizing cancer management among older adults. In a recent cost-utility analysis of the Canadian 5C randomized controlled trial (RCT), GAM appeared cost effective only in selective patients. We assessed the cost-utility of GAM plus usual care (UC) versus UC alone in older adults with cancer using a decision model and best available evidence from four international RCTs: GAIN, GAP70, INTEGERATE, and 5C.

**Methods:**

We conducted a model-based economic evaluation using pooled data from four international RCTs (GAIN, GAP70, INTEGERATE, and 5C), supplemented by additional evidence from the literature. Deterministic and probabilistic analyses were performed from the healthcare payer perspective, applying a six-month time horizon. The base case and main analyses used Canadian cost data. Sensitivity analyses included per-trial scenario analyses, one-year time horizon, and the use of USA costs. We reported healthcare costs per quality-adjusted life year (QALY) and the incremental net monetary benefit (INMB) using a CAD50,000(USD36,277) per QALY threshold.

**Results:**

The base case analysis using Canadian costs indicated that GAM was cost effective with an INMB of CAD1,117 (USD819) (95% credibility interval [CrI]: −CAD2,450 [−USD1,796], CAD5,035 [USD3,692]) and 70.7 percent probability of GAM being cost effective at a cost-effectiveness threshold of CAD50,000 (USD36,666) per QALY. Trial-specific results varied, with the GAP70 and INTEGERATE trials yielding positive INMB values (CAD2,635 [USD1932] and CAD2,886 [USD2,116], respectively), while 5C and GAIN resulted in negative INMB values (−CAD642 [−USD471] and −CAD268 [−USD196], respectively). Sensitivity analyses revealed that chemotherapy costs were the main driver of costs in both GAM and UC strategies.

**Conclusions:**

Evidence showed that GAM is generally cost effective. However, GAM’s cost effectiveness varied across trial scenarios, driven primarily by differences in chemotherapy costs. Future research should focus on identifying key GAM components that most effectively reduce severe toxicity, hospitalizations, and chemotherapy-related costs to optimize overall cost effectiveness.

